# Refractory immune thrombocytopenic purpura associated with IgM monoclonal gammopathy of undetermined significance: Successful treatment with tirabrutinib plus conventional therapies

**DOI:** 10.1002/jha2.423

**Published:** 2022-03-17

**Authors:** Ryosuke Naka, Hitomi Kaneko, Osamu Nagata, Kohei Tada, Masaharu Tashima, Chisato Mizutani, Kazunori Imada

**Affiliations:** ^1^ Department of Hematology Japanese Red Cross Osaka Hospital Osaka Japan

**Keywords:** IgM‐MGUS, refractory ITP, tirabrutinib

## Abstract

When immune thrombocytopenia (ITP) is secondary to malignant diseases, chemotherapy is expected to improve the platelet count (PC) as well. Herein, we report a case of a 72‐year‐old man with ITP refractory to standard therapies. IgM monoclonal gammopathy of undetermined significance (MGUS) was determined as an underlying disease. After bendamustine and rituximab (BR) therapy was found inadequately effective, tirabrutinib, a novel Bruton's tyrosine kinase inhibitor, was initiated, and the PC normalised subsequently. Surveillance of underlying diseases with which effective therapies are available may help manage refractory ITP, and IgM‐MGUS is potentially a targetable underlying disease with this newly available drug.

## 1 CASE

Immune thrombocytopenia (ITP) is an autoimmune disease, with the incidence of two to four cases per 100,000 person‐years worldwide [1]. Lymphoproliferative disorders (LPDs) are significant causes of secondary ITP, while other causes include drugs, infections, other autoimmune diseases, and other types of cancer. Among different LPDs, chronic lymphocytic leukaemia (CLL) [2], Hodgkin's lymphoma [3] and Waldenstrom macroglobulinemia (WM)[4] are well‐characterised etiologies for secondary ITP. ITP is reported to be complicated in 2.6% of monoclonal gammopathy of undetermined significance (MGUS) patients [5]. However, the clinical characteristics and optimal treatments of MGUS‐associated ITP are yet to be elucidated mainly due to the limited cases reported so far.

Corticosteroid is the definitive frontline therapy for ITP. Seventy to eighty per cent of patients show initial response [6], while a high relapse rate requires second‐line treatments, including rituximab, splenectomy and thrombopoietin receptor agonists (TRAs). Once refractory to these treatments, no more therapies are available as standard of care. Multiple drugs, including Bruton's tyrosine kinase inhibitors (BTKi), are now under investigation for refractory ITP patients. Targeting lymphoid cells to inhibit autoantibody production is regarded as a proof of concept. Hence, the drug initially developed for malignant lymphoma is potentially effective for refractory ITP, as in the case of rituximab. We herein report a case of refractory ITP with IgM‐MGUS, who was successfully treated with tirabrutinib, a newly developed BTKi approved for WM, lymphoplasmacytic lymphoma (LPL) and primary central nervous system lymphoma (PCNSL) in Japan.

**FIGURE 1:**
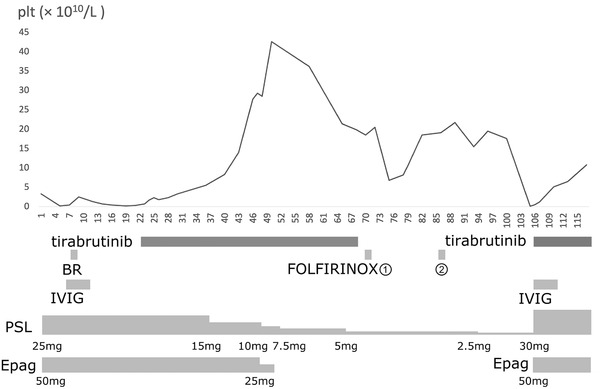
Time course of the platelet count. The treatments conducted are depicted below. The dose of tirabrutinib was stuck to 320 mg/body. Intravenous immunoglobulin (IVIG) was administered for 5 days at a dose of 400 mg/kg. FOLFIRINOX, the chemotherapy against pancreatic cancer, stands for folinic acid, fluorouracil, irinotecan and oxaliplatin

A 72‐year‐old Japanese male was diagnosed as primary ITP in a clinic 6 months prior to admission. The platelet count (PC) was 3.0 × 10^9^/L at initial diagnosis, when oral mucosal and gastrointestinal bleeding was complicated. Prednisolone (PSL), TRA, rituximab, danazol, in addition to intravenous immunoglobulin (IVIG), were introduced to achieve normal PC. TRA was switched from eltrombopag (Epag) to romiplostim(Romi) due to its inadequate effect, both administered at the maximum dose. Four months later, after PSL was gradually tapered from 40 to 20 mg; PC dropped to 4.0 × 10^9^/L. IVIG was again administered, and PC was stable as PSL was increased to 30 mg. He was unwilling to receive a splenectomy. PSL was again decreased to 20 mg 1 week prior to admission. He was transferred to our hospital for succeeding treatments, when PC was 33 × 10^9^/L (day 1, Figure 1). Thus PSL was increased to 25 mg, and Romi was again switched to Epag. The patient was hospitalised as PC dropped to 3.0 × 109/L on day 6. He was well appearing with normal vital signs. A physical examination revealed petechiae and ecchymosis in the limbs and oral mucosa. He was an otherwise healthy, retired businessman living with his wife. Other medications included acyclovir, vonoprazan fumarate, trimethoprim‐sulfamethoxazole, danazol and sodium risedronate hydrate.

**FIGURE 2:**
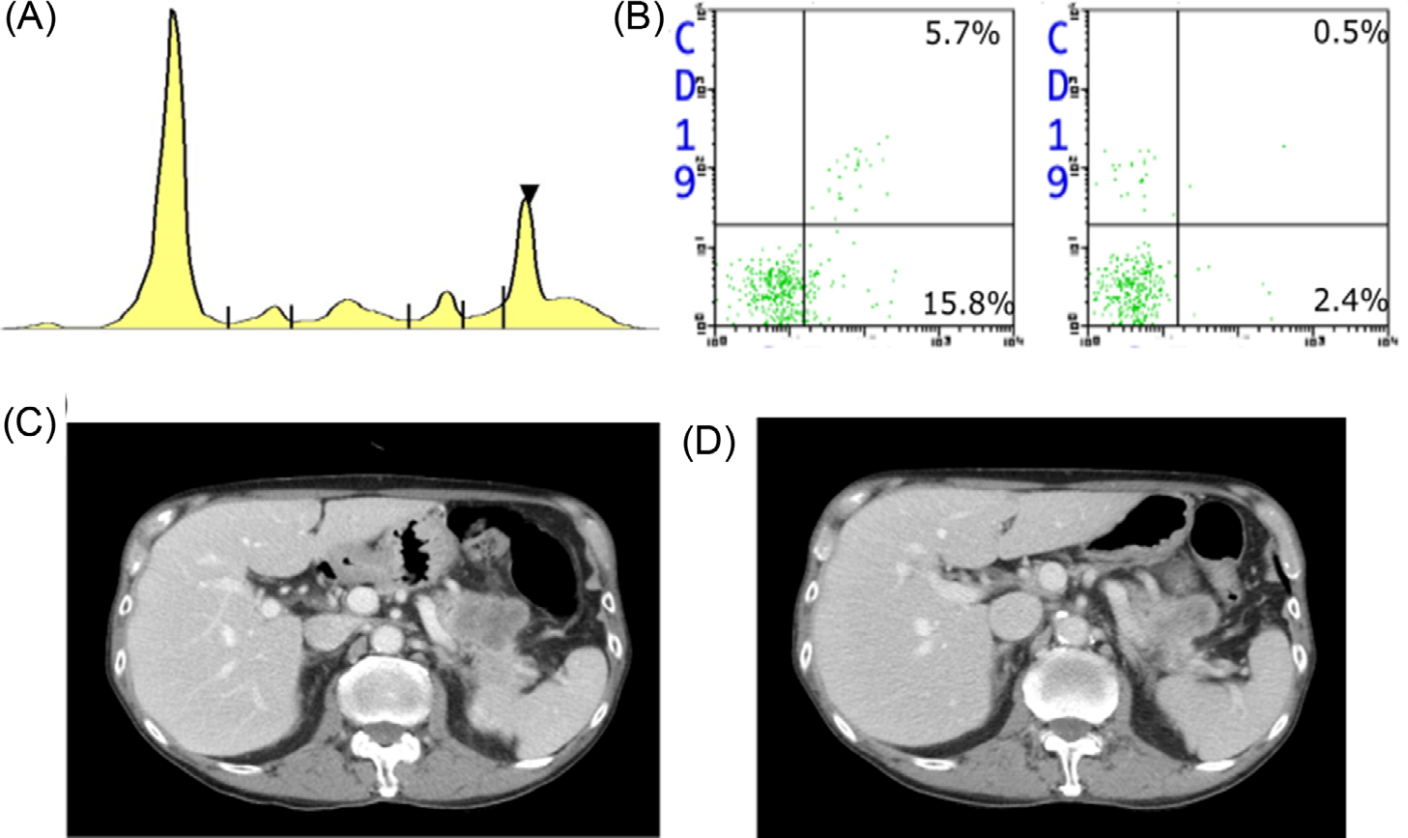
(A) Serum electrophoresis on day 1. (B) Flow cytometry analysis from bone marrow aspirate sample on day 1. Each dot represents lymphoid cells gated in the forward scatter (FSC)/side scatter (SSC) method. (C) Pancreatic lesion on day 6 by computed tomography (CT) imaging. (D) Pancreatic lesion on day 97

Given the poor response to standard therapies described above, the cause of thrombocytopenia was revised. Laboratory tests indicated an elevated level of IgM (1619 mg/dl), whereas IgG and IgA were within the normal range. Other laboratories included an average white blood cell count (11.9 × 10^9^/L), normal haemoglobin (14.5 g/L), an increased level of interleukin‐2 receptor level (827 U/ml) and a high titer of platelet‐associated IgG (85 ng/10^7^ cells). Serum electrolyte, renal and liver function tests were normal. Serum protein electrophoresis revealed M peak, which turned out to be IgM‐κ protein (Figure 2A). Abnormal cells were not apparent in a bone marrow aspiration, with mature plasma cells accounting for 0.6% of total nucleated cells. Flow cytometric analysis of bone marrow revealed a small population with immunoglobulin light chain restriction in the lymphocyte fraction (Figure 2B). Computed tomography did not show lymph node enlargement or splenomegaly. A large mass with poor contrast enhancement was identified in the pancreatic tail. Rapid recovery of PC was necessitated for histological diagnosis of the lesion.

We determined IgM‐MGUS as an underlying disease of refractory ITP. BR therapy with reduced intensity (bendamustine 50 mg/m^2^ and rituximab 375 mg/m^2^) was initiated on day 7. We continued to administer PSL (25 mg) and Epag (50 mg). Although IVIG showed a minimal transient response of PC (25 × 10^9^/L), it did not exceed 13 × 10^9^/L thereafter. Melaena and a rapid decrease in haemoglobin was observed on day 23, implying gastrointestinal bleeding. Platelet transfusion increased PC to 23 × 10^9^/L, and the haemorrhage resolved in a few days without further intervention. Diagnostic endoscopy was hampered by low PC. Although serum IgM was reduced to 1212 mg/dl on day 23, BR therapy had an inadequate effect on PC. Hence tirabrutinib (320 mg) was initiated as a second‐line therapy on day 24, with the informed consent of the patient and his family. PC gradually recovered and became normalised on day 46. PSL was tapered, and Epag was discontinued. PC remained normal for the following weeks.

Endoscopic ultrasound revealed the histological diagnosis of stage III pancreatic cancer. He then underwent chemotherapy (FOLFIRINOX) on day 70, when tirabrutinib was discontinued. On day 97, minimal response of pancreatic lesion was confirmed (Figure 2C,D). On day 105, PC suddenly dropped to 1.0 × 10^9^/L with no trigger identified, such as infection or vaccination. IVIG, tirabrutinib, PSL and Epag were immediately resumed. Serum IgM was lower than ever before (226 mg/dl). PC quickly recovered to 51 × 10^9^/L on day 110, without lethal complications. On day 117, he was in outpatient follow‐up, preparing for future treatment against pancreatic cancer.

ITP is associated with various types of malignant diseases, especially LPDs. When ITP is found refractory to standard therapies, the diagnosis should be reassessed [7]. In our patient, serum IgM level and protein electrophoresis helped diagnose IgM‐MGUS. Although pancreatic cancer was concurrently diagnosed, we speculated that IgM‐MGUS was more associated with ITP considering its high frequency in WM. Since ITP was refractory to standard therapies, chemoimmunotherapy against IgM‐MGUS was conducted. To the best of our knowledge, this is the first case to report successful treatment of IgM‐MGUS‐associated ITP with targeted therapy against IgM‐MGUS.

WM is an LPL with IgM paraprotein, which has an indolent clinical course. ITP is complicated in 3.8% of WM patients [5], besides well‐known paraneoplastic syndromes such as haemolytic anaemia, peripheral neuropathy and cold agglutinins [4]. Chemotherapy is indicated when the disease becomes symptomatic, and 42% of patients start treatment due to autoimmune cytopenia [8]. IgM‐MGUS, a precancerous lesion of WM, on the other hand, has no consensus indication or regimen of the treatment. A case report showed that peripheral neuropathy was mitigated after treatment of IgM‐MGUS [9]. Our case has novelty in terms of targeting IgM‐MGUS when managing associated ITP. As multiple myeloma and MGUS are occasionally concomitant with ITP [5, 10], M protein should be investigated when managing refractory ITP.

BTKi is expected to have both anti‐tumour efficacy and immunosuppressive activity, as BTK plays a crucial role in the development and functioning of B lymphocytes. Ibrutinib, a first‐in‐class drug, approved for lymphocytic malignancy such as CLL, is associated with improvement of CLL‐related ITP [11]. Tirabrutinib is a second‐generation, highly selective BTKi, with approval in Japan for PCNSL, WM and LPL [12, 13]. PC resolved quickly after induction of this drug twice; on days 22 and 105. It is noteworthy that the effect of tirabrutinib alone cannot be evaluated in this single case. The investigation with a larger number of patients would be warranted. When PC dramatically dropped on day 105, serum IgM was within normal range, indicating that IgM‐MGUS was under control while ITP relapsed. One possible explanation is that tirabrutinib may have an independent immunomodulatory effect to suppress autoimmune platelet destruction rather than inhibiting tumour cell growth of IgM‐MGUS. Indeed rilzabrutinib, another BTKi, is currently under investigation for relapsed or refractory ITP.

Bleeding is a significant effect of BTKi. BTK comprises a crucial pathway of platelet activation. Tirabrutinib was associated with a grade 1 or 2 bleeding event in 5.6%–11.1% of patients in clinical trials [12, 13]. In our case, gastrointestinal bleeding was confirmed on day 25, the next day after starting tirabrutinib. However, their causal association was not evident because the bleeding might have occurred on day 23, inferred by serum blood urea nitrogen ratio over creatinine. We still argue that BTKi including tirabrutinib should be administered with bleeding events carefully monitored.

In summary, this is the first case to describe IgM‐MGUS‐associated ITP successfully treated with BTKi in addition to conventional therapies. Our case implies that IgM‐MGUS can be underestimated but targetable underlying disease of ITP. The potential efficacy of BTKi for refractory ITP associated with IgM‐MGUS or other B‐cell malignant diseases should be further evaluated with a larger number of patients.

## FUNDING INFORMATION

The authors received no specific funding for this work

## CONFLICT OF INTEREST

The authors declare that there is no conflict of interest that could be perceived as prejudicing the impartiality of the research reported.
